# Tetrahydropalmatine improves mitochondrial function in vascular smooth muscle cells of atherosclerosis *in vitro* by inhibiting Ras homolog gene family A/Rho-associated protein kinase-1 signaling pathway

**DOI:** 10.1515/med-2024-1059

**Published:** 2025-03-17

**Authors:** Ke Ding, Qiying Bao, Jiaqi He, Jiahong Wang, Hui Wang

**Affiliations:** Department of Pharmacy, The First Affiliated Hospital of Zhejiang Chinese Medical University, No. 54, Youdian Road, Hangzhou, 310006, Zhejiang, China; Department of Pharmacy, Hangzhou Fuyang Hospital of TCM Orthopedics, Hangzhou, 311499, Zhejiang, China; Traditional Chinese Medicine Dispensary, The First Affiliated Hospital of Zhejiang Chinese Medical University, Hangzhou, 310006, Zhejiang, China; School of Pharmaceutical Sciences, Zhejiang Chinese Medical University, No. 548, Binwen Road, Hangzhou, 310053, Zhejiang, China

**Keywords:** tetrahydropalmatine, atherosclerosis, VSMCs, mitochondrial function, RhoA/ROCK1 pathway

## Abstract

**Background:**

Tetrahydropalmatine (THP) regulates mitochondrial function in vascular smooth muscle cells (VSMCs) to prevent or alleviate atherosclerosis (AS), with unclear specific mechanism.

**Methods:**

AS models were constructed by oxidized low-density lipoprotein (ox-LDL)-treated VSMCs. Cell counting kit-8 for cell viability, wound scratch assay for cell migration, and flow cytometry for cell cycle, intracellular reactive oxygen species, and mitochondrial membrane potential (MMP) were performed. Malondialdehyde (MDA) and superoxide dismutase (SOD) levels by biochemical kits, oxygen consumption rate (OCR) by seahorse apparatus, apoptosis by terminal deoxynucleotidyl transferase-mediated dUTP nick end labeling assay (TUNEL) staining, and apoptosis-related expression by western blot were detected. Ras homolog gene family A/Rho-associated protein kinase-1 (RhoA/ROCK1) levels were measured by western blot and ELISA. The RhoA agonist, U46619, was employed to validate mechanism of THP.

**Results:**

THP suppressed cell cycle progression and cell migration whereas alleviating cell viability and oxidative stress, as reduced MDA and enhanced SOD levels in ox-LDL-incubated VSMCs. THP protected mitochondrial function by higher MMP levels and OCR values. Additionally, THP decreased TUNEL-positive cells, Bax, Caspase-3, RhoA, ROCK1, and osteopontin expression, while increased Bcl-2 and smooth muscle myosin heavy chain levels. Furthermore, U46619 intervention antagonized effects of THP.

**Conclusion:**

THP improved mitochondrial function in VSMCs of AS by inhibiting RhoA/ROCK1 signaling pathway.

## 1 Introduction

Cardiovascular disease (CVD) caused by atherosclerosis (AS) is the main cause of death and disability of the public [1]. According to the American Heart Association reports, approximately 17.3 million people die from CVD each year all over the world [2]. Arterial remodeling (AR) is the main pathological basis of AS, and vascular smooth muscle cells (VSMCs) are the main cells in the middle layer of artery wall, and at the same time, VSMCs participate in AR [3]. Dedifferentiated VSMCs lose their contractility and transform into different phenotypes, including synthetic, secretory, proliferative, and migratory phenotypes which play critical roles in the pathogenesis process of AR [4]. As the primary place of eukaryotic oxidation and energy conversion, the mass and quality of mitochondria are necessary for maintaining the metabolic process of VSMCs [5]. Impaired mitochondrial function can cause dysfunction of signal transduction and metabolism and may promote the instability of atherosclerotic plaques. Mitochondrial dysfunction impairs the production of adenosine triphosphate (ATP), increases superoxide production, causes oxidative stress damage, and induces subsequent apoptosis of VSMCs [6]. Consequently, reducing oxidative stress damage and maintaining mitochondrial function play a role in the preventive and therapeutic effects of AS.

Ras homolog gene family (Rho)-proteins are widely distributed in mammalian tissue cells and involved in the cell proliferation, apoptosis, and gene expression [7]. Rho-proteins are divided into three subtypes, among which RhoA has been studied most deeply. The downstream Rho-associated protein kinase (ROCK) is one of the earliest and most thoroughly studied downstream effects of RhoA [8,9]. RhoA/ROCK1 signaling pathway has participated in the occurrence and development of multiple CVD in multiple manners [10], such as AS, high blood pressure, heart failure, hemangioma, and reperfusion injury of blood deficiency. This signaling pathway is involved in mitochondrial dysfunction [11]. Therefore, the suppression of RhoA/ROCK1 signaling pathway has important significance in preventing and treating AS.

At present, AS is regarded as a cholesterol storage disease, primarily prevented through the use of statins, such as atorvastatin and rosuvastatin to control blood lipid levels. However, the efficacy of statins is limited and long-term use or large dosage may lead to rhabdomyolysis [12,13]. So, we turned our attention to the natural medicine. *Corydalis yanhusuo* W.T.Wang has many pharmacological effects [14], such as promoting blood circulation, dilating blood vessels, anti-thrombus, reducing blood pressure, anti-inflammatory, antioxidant, and analgesia, etc. As the main component of *Corydalis yanhusuo* W.T.Wang, tetrahydropalmatine (THP) has versatile pharmacologic activities, including antiarrhythmia, improving hemodynamics and decreasing blood lipids [15]. It has been revealed that THP protects the cardiovascular system by reducing blood pressure and alleviating myocardial infarction [16]. The current research works mainly focus on the anti-oxidative stress and anti-apoptosis ability of THP in CVD [17], but further molecular mechanisms are still unknown.

Therefore, we aim to clarify the role of THP on mitochondrial function by an *in vitro* model of AS constructed from oxidized low-density lipoprotein (ox-LDL)-induced VSMCs, and to explore effects of THP on regulation of the RhoA/ROCK1 signaling pathway in ox-LDL-induced VSMCs by utilizing the RhoA agonist U46619 (9,11-Methanoepoxy PGH2, an analogue of Thromboxane A2), providing scientific basis for the clinical application of THP in AS treatment, and facilitating THP as a potential drug candidate for AS treatment.

## 2 Methods

### 2.1 Chemicals and reagents

THP (Y39962) was obtained from Shanghai yuanye Bio-Technology Co., Ltd (China). Cell counting kit-8 (CCK-8, C0039), reactive oxygen species (ROS) detection kit, Mitochondrial membrane potential (MMP) detection kit, One-step terminal deoxynucleotidyl transferase-mediated dUTP nick end labeling assay (TUNEL) Cell Apoptosis Detection Kit (Red fluorescence), and BCA protein assay kit (pc0020) were provided by Beyotime Biotechnology (China). Cell malondialdehyde (MDA) assay kit (A003-4-1) and superoxide dismutase (SOD) assay kit (A001-3-1) were purchased from Nanjing Jiancheng Bioengineering Institute (China). RhoA enzyme linked immunosorbent assay (ELISA) kit (MM-60097H2) and ROCK1 ELISA kit (MM-15121H2) were purchased from Jiangsu Meimian Industrial Co., Ltd (China). Seahorse XF real-time ATP generation rate assay kit (103325-100) was provided by Agilent Technologies, Inc (USA). Propidium (PI)/RNase Staining Buffer (550825) was purchased from BD Pharmingen™. RhoA Antibody (AF6352), ROCK1 Antibody (AF7016), α-smooth muscle actin (α-SMA) Antibody (BF9212), smooth muscle myosin heavy chain (SM-MHC) Antibody (DF8344), Osteopontin (OPN) Antibody (AF0227), B-cell lymphoma-2 (Bcl-2) Antibody (AF6139), Bcl2-associated X (Bax) Antibody (AF0120), Caspase-3 Antibody (AF6311) were purchased from Affinity Biosciences (USA). GAPDH Antibody (10494-1-AP) was provided by Proteintech (USA).

### 2.2 Cell culture and grouping

Human aortic VSMCs (HA-VSMCs, CL-0517) were purchased from Procell Life Science&Technology Co., Ltd (China). VSMCs were cultured in a medium containing 2% FBS, 100 U/mL penicillin-streptomycin, and 1% smooth muscle cell growth factor in a cell incubator (5% CO_2_, 37°C). The medium was changed every 3 days. The cells were divided into five groups: Control group, ox-LDL (50 μg/mL) group, and ox-LDL (50 μg/mL) + THP group (5, 10, 20 µg/mL THP) to determine the optimal THP dosage. Then, three groups were used: Control group, ox-LDL (50 μg/mL) group, and ox-LDL (50 μg/mL) + THP (10 µg/mL) group to observe the effects of THP on cell cycle, cell migration, apoptosis, and RhoA/ROCK1 pathway-related expression of VSMCs. Four groups were used: Control group, ox-LDL (50 μg/mL) group, THP (10 µg/mL) group, and ox-LDL (50 μg/mL) + THP (10 µg/mL) group to detect the oxidative stress and mitochondrial function in VSMCs. Finally, four groups were used: Control group, ox-LDL (50 μg/mL) group, ox-LDL (50 μg/mL) + THP (10 µg/mL) group, and ox-LDL (50 μg/mL) + THP (10 µg/mL) + U46619 (a RhoA pathway agonist, 100 nM) [18,19] group (U46619 group) to elucidate the mechanisms of THP.

### 2.3 Cell proliferation assay

VSMCs were inoculated into 96-well plates. Cell viability with or without ox-LDL and THP (5, 10, 20 µg/mL) intervention was detected using CCK-8. In brief, according to the instruction, after 24 h of cell culture, 10 μL CCK-8 solution was added to each well and incubated for 2 h in the cell incubator (5% CO_2_, 37°C). The absorbance was determined by a microplate reader (CMaxPlus, MD, USA) at 450 nm, and then the cell viability was calculated according to the formula of CCK-8.

### 2.4 Measurement of cell cycle

VSMCs were collected and washed by phosphate buffer saline (PBS), discarding the supernatant after centrifugation. PI/RNase staining buffer and permeabilization solution were added to resuspend cells followed by incubation for 30 min at room temperature (25°C) away from light. A flow cytometry (C6, BD, USA) was used to detect the cell cycle.

### 2.5 Cell migration assay

VSMCs were inoculated into 6-well plates and starved for 24 h after the wells were fully covered with cells. The pipette gun head was used to vertically scratch the 6‐well plates. VSMCs were cleaned with PBS to wash away the cells under the scratches, followed by ox-LDL (50 µg/mL) and THP (10 µg/mL) intervention. After 48 h culture, the wound scratch healing was examined and photographed via an optical microscope (AE2000, Motic, China). The wound scratch healing rate of VSMCs was analyzed and calculated by Image J.

### 2.6 Determination of MDA and SOD

VSMCs were digested and collected by centrifuge, and the pre-cooled cell lysate was added, cracked on ice, and centrifuged to take the supernatant. The changes in MDA and SOD levels in VSMCs were measured according to kit instructions. The microplate reader was used to test the absorbance values at 530 nm (MDA) and 450 nm (SOD).

### 2.7 ROS detection

The ROS content of VSMCs was detected using a 2′,7′-Dichlorofluorescein diacetate (DCFH-DA) fluorescent probe. After intervention of ox-LDL and THP, 500 μL diluted DCFH-DA (10 μM) was added and co-incubated at 37°C for 20 min. Whereafter, after fixing, permeation, and DAPI incubation, the fluorescence intensity was examined and pictured under an inverted fluorescence microscope (Ts2-FC, Nikon, Japan) at 488/525 nm.

### 2.8 MMP detection

MMP of VSMCs was detected by a JC-1 fluorescent probe. In brief, 500 μL DCFH-DA (10 μM) was added to each group well and co-cultured at 37°C. Afterwards, the residual JC-1 staining was washed with PBS. After the fixing, permeation, and DAPI incubation, the red fluorescence and green fluorescence were obtained and photographed under the inverted fluorescence microscope at 490/530 nm and 525/590 nm [20].

### 2.9 Mitochondrial respiratory function

The measurement of oxygen consumption rate (OCR), including basal OCR, ATP-linked OCR, and maximal OCR was performed by an XF96 extracellular flux analyzer (Seahorse Bioscience, Agilent Technologies) [21]. The VSMCs were inoculated at a density of 10^4^/well into the 96-well of Seahorse XF96 cell culture microplates. Following overnight incubation in a cell culture incubator (5% CO_2_, 37°C), the medium was discarded and 120 µL of XF assay medium, i.e., low-buffered bicarbonate-free Dulbecco’s modified eagle medium (DMEM) (pH 7.4) was added for incubation in a CO_2_-free 37°C incubator for 1 h. Then, the detection solution was injected in sequential order, including 1 µmol/L of oligomycin, 1 µmol/L of carbonyl cyanide phospho-(*p*)-trifluoromethoxy phenylhydrazone (FCCP, a proton gradient uncoupler), and 0.5 µmol/L of rotenone/antimycin. The OCR value before oligomycin injection was the basal OCR. Oligomycin injection inhibited ATP synthase, leading to a reduction in mitochondrial respiration and OCR value, with this reduction in OCR being associated with cellular ATP production, as ATP-linked OCR. The increase in cellular oxygen consumption after the second FCCP injection was the maximal OCR. Rotenone/antimycotic injection turned off mitochondrial respiration and enabled the calculation of non-mitochondrial respiration driven by processes outside the mitochondria. The determination of cellular protein content was done using a MicroBCA kit (Thermofisher Scientific, Waltham, MA USA) and OCR values were normalized, expressed as pMoles/min/µg protein.

### 2.10 Cell apoptosis assay by TUNEL staining

After fixing and permeation, VSMCs were incubated with TUNEL testing fluid at 37°C for 60 min away from light. The images were assessed and photographed under the inverted fluorescence microscope.

### 2.11 ELISA assay

The supernatant liquid of VSMCs in each group was collected, and RhoA and ROCK1 levels were detected using ELISA kits according to the manufacturers’ instructions. The absorbance value of all samples was detected by using the microplate reader at 450 nm.

### 2.12 Western blot

The total protein of VSMCs was extracted and 10% sodium dodecyl sulfate-polyacrylamide gel electrophoresis was prepared to separate proteins of different molecular weights, followed by transfer of the protein samples to polyvinylidene fluoride (PVDF) membranes electrophoretically. After rinsing with Tris buffered saline with Tween-20 (TBST), PVDF membranes were blocked with 5% BSA for 1.5 h at room temperature (25°C) and immediately incubated with primary antibodies against RhoA, ROCK1, α-SMA, SM-MHC, OPN, Bcl-2, Bax, Caspase-3 in a dilution of 1:1,000 and glyceraldehyde-3-phosphate dehydrogenase in a dilution of 1:10,000 overnight at 4°C. The next day, membranes were incubated with secondary antibody (1:6,000 dilution) for 1.5 h at room temperature, followed by washing with TBST thrice. Ultra-enhanced chemiluminescence chemiluminescent solution was prepared of a mixture of liquid A and liquid B and images were captured using a chemiluminescence apparatus (610020-9Q, Shanghai Qinxiang Scientific Instrument Co., Ltd, China).

### 2.13 Statistical analysis

All statistics were presented as the mean value ± standard deviation (mean value ± SD) and statistical analyzes are performed using SPSS 20.0. Comparisons between multiple groups were performed by One-Way ANOVA and the Tukey’s post-test was used for further pound-to-group comparison. Significance level *α* = 0.05. *P* < 0.05 was considered statistically significant.


**Ethic approval and consent to participate:** Not applicable.
**Consent for publication:** Not applicable.

## 3 Results

### 3.1 THP regulates cell viability, cell cycle, and cell migration in ox-LDL-incubated VSMCs

**Figure 1:**
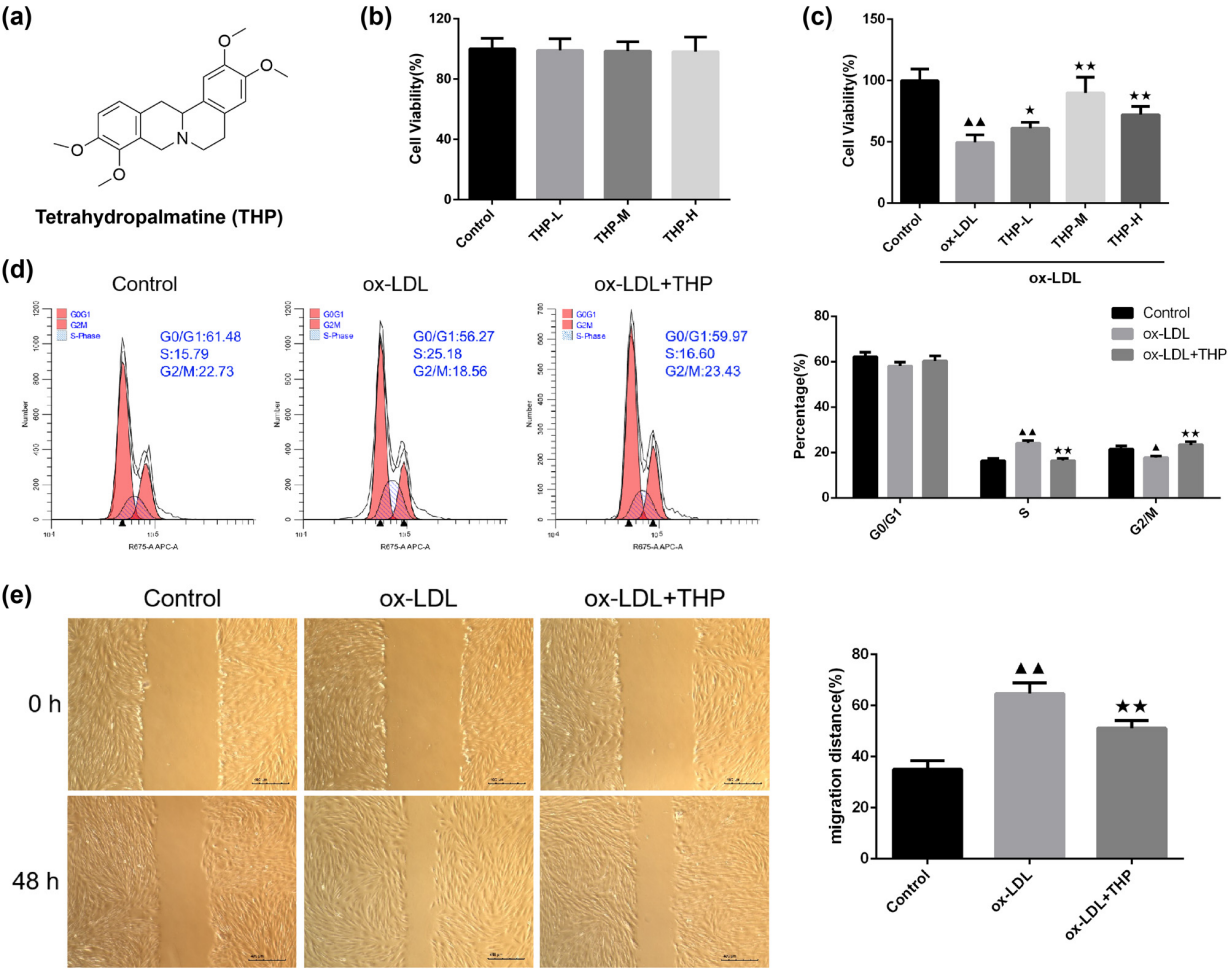
THP regulates cell viability, cell cycle, and cell migration in ox-LDL-incubated VSMCs. (a) The chemical structure formula of THP. (b) and (c) Detection of cell viability of THP in VSMCs and in ox-LDL-incubated VSMCs was done by CCK8 assay (*n* = 6). (d) Cell cycle of THP on ox-LDL-incubated VSMCs was tested by flow cytometry (*n* = 3). (e) The measurement of cell migration in VSMCs was carried out using cell scratching assay (*n* = 3, Scale bar = 400 μm). The above data are presented as mean value ± SD. Compared to the Control group, ^▲^ *P* < 0.05 and ^▲▲^ *P* < 0.01; Compared to the ox-LDL group, ^★^ *P* < 0.05 and ^★★^ *P* < 0.01. Note: THP: tetrahydropalmatine; ox-LDL: oxidized low-density lipoprotein; VSMCs: vascular smooth muscle cells.

The chemical structure formula of THP was demonstrated in Figure 1a. CCK8 assay was employed to detect the cell viability of VSMCs with different concentrations of THP (5, 10, 20 µg/mL) and cell viability of VSMCs induced by ox-LDL (50 µg/mL) with 5, 10, 20 µg/mL of THP (Figure 1b and c). The THP treatment at 5, 10, 20 µg/mL caused no difference to cell viability in comparison with control VSMCs (Figure 1b, *P* > 0.05). In Figure 1c, there was decreased cell viability after ox-LDL intervention to VSMCs (*P* < 0.01), whereas higher cell viability after co-culture with THP (5, 10, 20 µg/mL) for 24 h in ox-LDL-incubated VSMCs (*P* < 0.01 or *P* < 0.05) was observed. According to the above results, 10 µg/mL of THP intervention was applied for subsequent detection.

The detection of cell cycle was made by flow cytometry as displayed in Figure 1d. There is no significant difference in the number of VSMCs in G0/G1 phase in ox-LDL group compared with the Control group; the number of VSMCs increased in S phase (*P* < 0.01) and decreased in G2/M phase (*P* < 0.05). In comparison with the ox-LDL group, ox-LDL + THP group demonstrated the smaller number of VSMCs in S phase (*P* < 0.01) and higher number of VSMCs in G2/M phase (*P* < 0.01).

The measurement of cell migration was done with cell scratching assay (Figure 1e). The 48-h mobility of VSMCs in ox-LDL group is elevated than that of the Control group (*P* < 0.01). There was reduced 48-h mobility of VSMCs in ox-LDL + THP group than in ox-LDL group (*P* < 0.01).

### 3.2 THP decreases oxidative stress in ox-LDL-induced VSMCs

**Figure 2:**
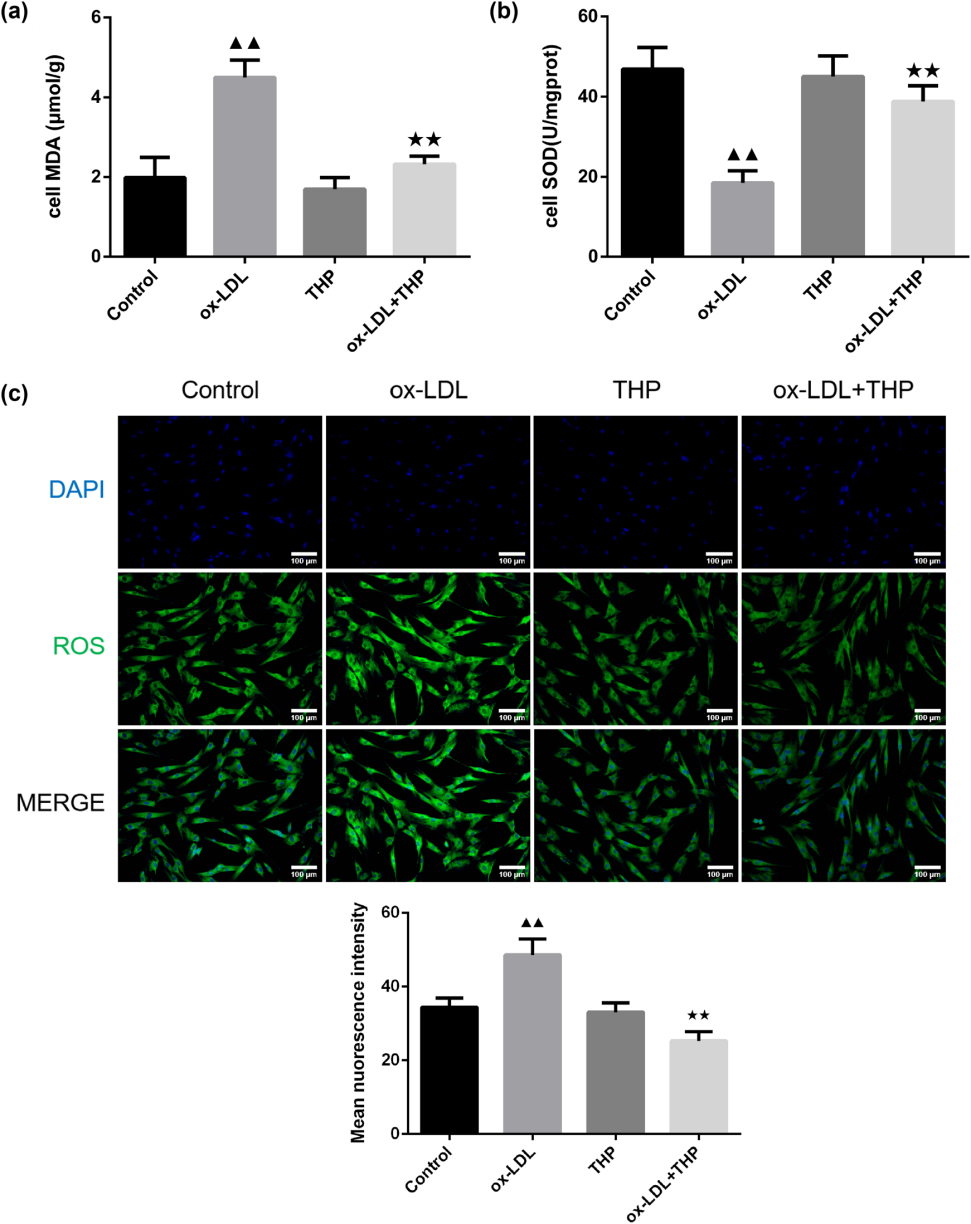
THP decreases oxidative stress in ox-LDL-induced VSMCs. (a) Effects of THP on MDA content of ox-LDL-incubated VSMCs (*n* = 6). (b) Effects of THP on SOD activity of ox-LDL-incubated VSMCs (*n* = 6). (c) Effects of THP on ROS content of ox-LDL-incubated VSMCs were detected by using DCFH-DA fluorescent probe (*n* = 3, Scale bar = 100 μm). The above data are presented as mean value ± SD. Compared to the Control group, ^▲▲^ *P* < 0.01; Compared to the ox-LDL group, ^★★^ *P* < 0.01. Note: THP: tetrahydropalmatine; ox-LDL: oxidized low-density lipoprotein; VSMCs: vascular smooth muscle cells; MDA: malondialdehyde; SOD: superoxide dismutase; ROS: reactive oxygen species; DCFH-DA: 2′,7′-Dichlorofluorescein diacetate.

Determination of MDA and SOD levels was carried out according to instructions of the corresponding kits in Figure 2a and b. The ox-LDL intervention led to higher MDA and lower SOD levels than those of Control group (*P* < 0.01). The cells in ox-LDL + THP group exhibited decreased MDA whereas enhanced SOD levels than in ox-LDL group (*P* < 0.01).

Additionally, the detection of ROS content was performed using the DCFH-DA fluorescent probe (Figure 2c). There was enhanced fluorescence intensity, that is, higher ROS content in ox-LDL-induced VSMCs in comparison to control cells (*P* < 0.01). Following further THP treatment the fluorescence intensity of ox-LDL-induced VSMCs reduced, indicating lowered ROS content (*P* < 0.01).

### 3.3 THP causes increased MMP in ox-LDL-treated VSMCs

**Figure 3:**
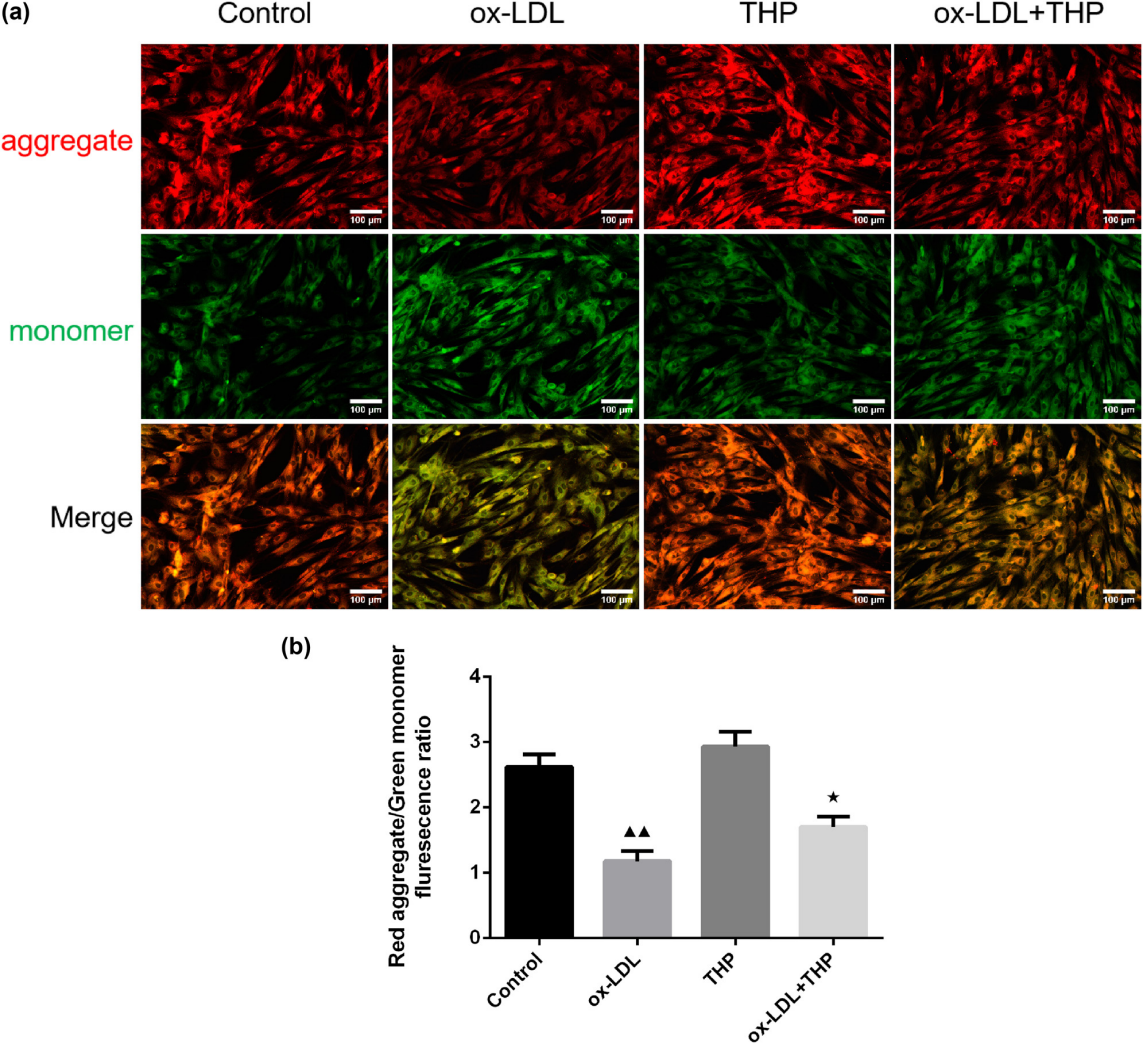
THP causes increased MMP in ox-LDL-treated VSMCs. (a) and (b) The JC-1 fluorescent probe was employed to examine the MMP of VSMCs (Scale bar = 100 μm). The above data are presented as mean value ± SD, *n* = 3. Compared to the Control group, ^▲▲^ *P* < 0.01; Compared to the ox-LDL group, ^★^ *P* < 0.05. Note: THP: tetrahydropalmatine; ox-LDL: oxidized low-density lipoprotein; VSMCs: vascular smooth muscle cells; MMP: mitochondrial membrane potential.

The JC-1 fluorescent probe was employed to examine the MMP of VSMCs as shown in Figure 3a and b. The ox-LDL group exhibited lower MMP than that of Control group (*P* < 0.01), whereas higher MMP in ox-LDL + THP group than in ox-LDL group (*P* < 0.05).

### 3.4 THP protects mitochondrial respiratory function in ox-LDL-incubated VSMCs

**Figure 4:**
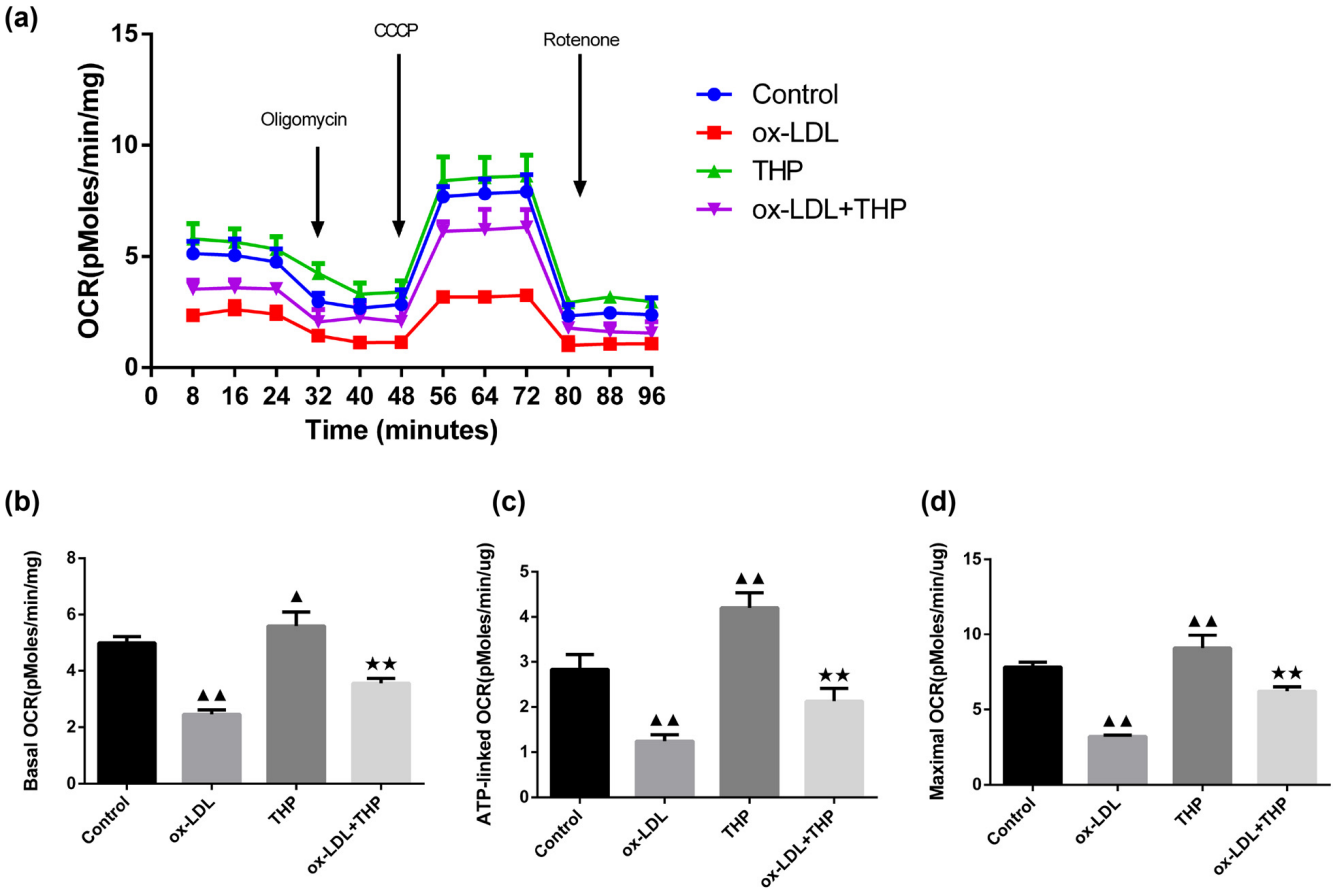
THP protects mitochondrial respiratory function in ox-LDL-incubated VSMCs. (a)–(d) The analysis of mitochondrial respiratory function was performed by test of extracellular OCR changes over 96 min, basal OCR, ATP-linked OCR, and maximal OCR using an XF96 extracellular flux analyzer. The above data are presented as mean value ± SD, *n* = 6. Compared to the Control group, ^▲^ *P* < 0.05 and ^▲▲^ *P* < 0.01; Compared to the ox-LDL group, ^★★^ *P* < 0.01. Note: THP: tetrahydropalmatine; ox-LDL: oxidized low-density lipoprotein; VSMCs: vascular smooth muscle cells; ATP: adenosine triphosphate; OCR: oxygen consumption rate.

The analysis of mitochondrial respiratory function was performed by test of extracellular OCR changes over 96 min, basal OCR, ATP-linked OCR, and maximal OCR using an XF96 extracellular flux analyzer (Figure 4a–d). The basal OCR, ATP-linked OCR, and maximal OCR values of ox-LDL group demonstrated a reduction than those of Control group (*P* < 0.01), whereas an increase in basal OCR, ATP-linked OCR, and maximal OCR values occurred in THP group (*P* < 0.01 or *P* < 0.05). The ox-LDL + THP group demonstrated elevated basal OCR, ATP-linked OCR, and maximal OCR values than those of ox-LDL group (*P* < 0.01).

### 3.5 THP reduces cell apoptosis in ox-LDL-induced VSMCs

**Figure 5:**
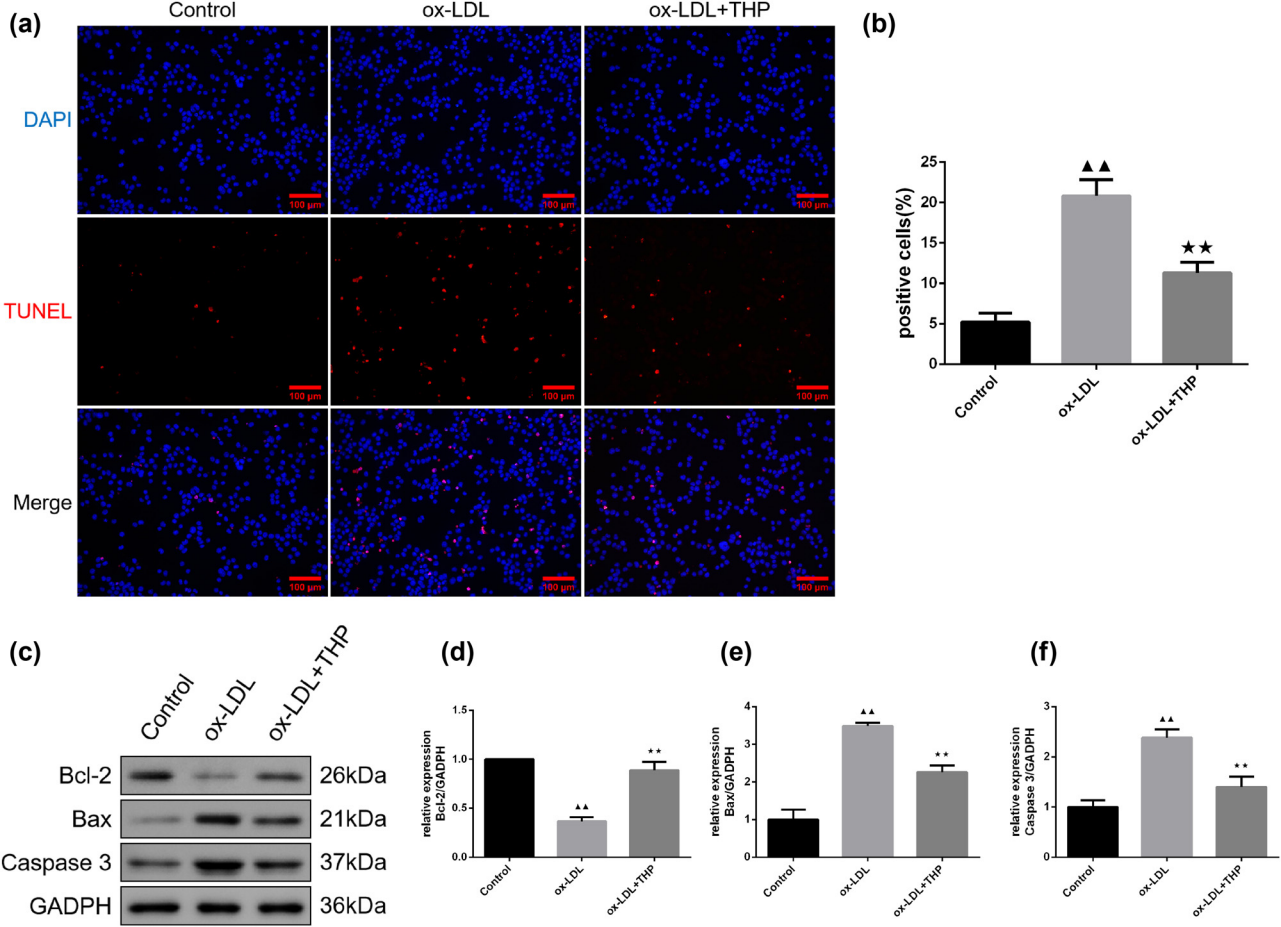
THP reduces cell apoptosis in ox-LDL-induced VSMCs. (a) and (b) Measurement of cell apoptosis was made by TUNEL staining (Scale bar = 100 μm). (c)–(f) Western blot assay was utilized to detect the apoptosis-related protein expression in VSMCs. The above data are presented as mean value ± SD, *n* = 3. Compared to the Control group, ^▲▲^ *P* < 0.01; Compared to the ox-LDL group, ^★★^ *P* < 0.01. Note: THP: tetrahydropalmatine; ox-LDL: oxidized low-density lipoprotein; VSMCs: vascular smooth muscle cells; Bcl-2: B-cell lymphoma-2; Bax: Bcl2-associated X.

Measurement of cell apoptosis was made by TUNEL staining in Figure 5a and b. There were elevated positive cell rate in ox-LDL-induced VSMCs than in Control group (*P* < 0.01), indicating enhanced apoptosis. Compared to ox-LDL group, positive cell rate reduced in ox-LDL + THP group, indicating inhibited apoptosis (*P* < 0.01).

Western blot assay was utilized to detect the apoptosis-related protein expression in VSMCs (Figure 5c–f). The ox-LDL-induced VSMCs demonstrated lowered Bcl-2 expression, whereas higher Bax and Caspase-3 expressions than that of Control group (*P* < 0.01) were demonstrated. In addition, the Bcl-2 expression of ox-LDL + THP group increased, with reduced Bax and Caspase-3 expression than in ox-LDL group (*P* < 0.01).

### 3.6 THP inhibits RhoA/ROCK1 signaling pathway-related protein expression and regulates phenotypic switching proteins in ox-LDL-treated VSMCs

**Figure 6:**
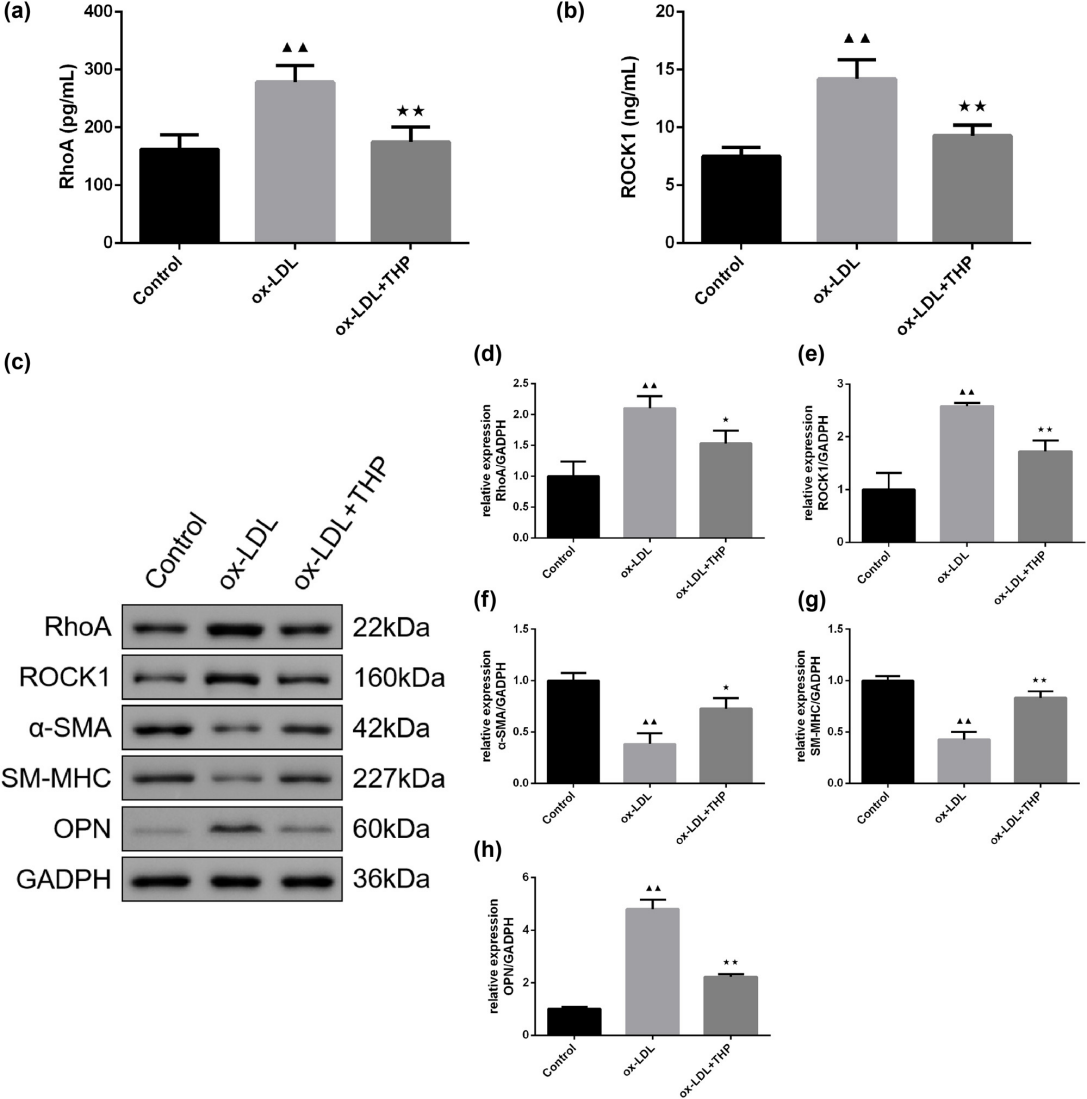
THP inhibits RhoA/ROCK1 signaling pathway-related protein expression and regulates phenotypic switching proteins in ox-LDL-treated VSMCs. (a) and (b) ELISA assay was employed for examination of RhoA and ROCK1 contents in ox-LDL-incubated VSMCs (*n* = 6). (c)–(h) Measurement of RhoA, ROCK1, α-SMA, SM-MHC, and OPN expression in VSMCs was made by western blot assay (*n* = 3). The above data are presented as mean value ± SD. Compared to the Control group, ^▲▲^ *P* < 0.01; Compared to the ox-LDL group, ^★^ *P* < 0.05 and ^★★^ *P* < 0.01. Note: THP: tetrahydropalmatine; ox-LDL: oxidized low-density lipoprotein; VSMCs: vascular smooth muscle cells; RhoA: Ras homolog gene family A; ROCK: Rho-associated protein kinase; α-SMA: α-smooth muscle actin; SM-MHC: smooth muscle myosin heavy chain; OPN: osteopontin.

The detection of RhoA and ROCK1 levels was carried out by ELISA assay (Figure 6a and b). The THP treatment reversed the increase in RhoA and ROCK1 levels in ox-LDL-treated VSMCs (*P* < 0.01). Moreover, expression of RhoA/ROCK1 signaling pathway-related proteins and phenotypic switching proteins was examined by western blot assay in Figure 6c–h. The ox-LDL intervention caused higher RhoA, ROCK1, and OPN protein expression and lower α-SMA and SM-MHC expression in VSMCs than that of Control group (*P* < 0.01) (*P* < 0.01). With the treatment of THP in ox-LDL-treated VSMCs, the protein expression of RhoA, ROCK1, and OPN were decreased (*P* < 0.01 or *P* < 0.05), while that of α-SMA and SM-MHC were promoted (*P* < 0.01).

### 3.7 THP reduces oxidative stress in ox-LDL-incubated VSMCs via inhibition of RhoA/ROCK1 signaling pathway

**Figure 7:**
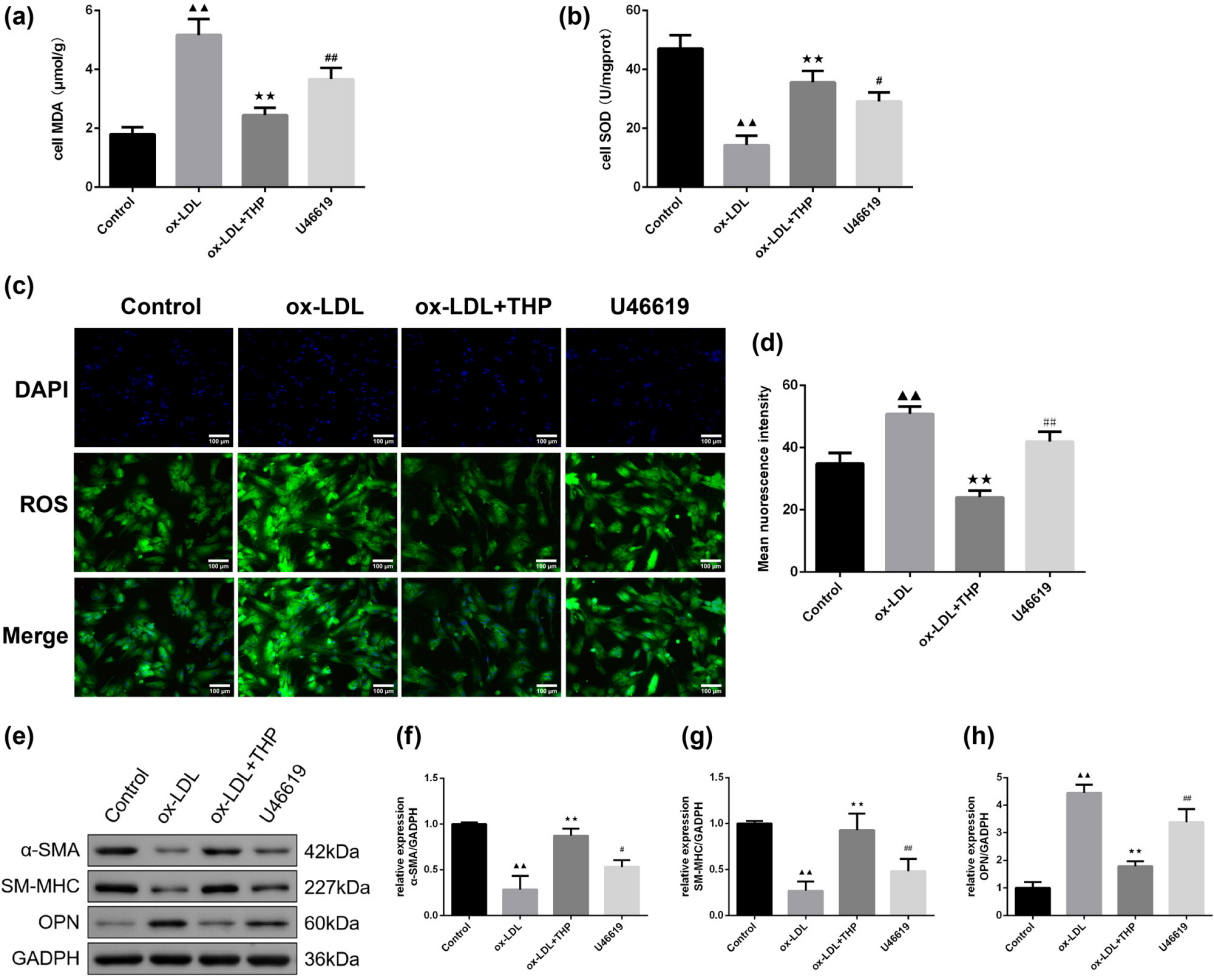
THP reduces oxidative stress in ox-LDL-incubated VSMCs via inhibition of RhoA/ROCK1 signaling pathway. (a) and (b) The content of MDA and activity of SOD in cell supernatant (*n* = 6). (c) and (d) Detection of ROS content was made by the DCFH-DA fluorescent probe (*n* = 3, Scale bar = 100 μm). (e)–(h) The phenotypic switching protein (α-SMA, SM-MHC, OPN) expression was tested using western blot assay (*n* = 3). The above data are presented as mean value ± SD. Compared to the Control group, ^▲▲^ *P* < 0.01; Compared to the ox-LDL group, ^★★^ *P* < 0.01; Compared to the ox-LDL + THP group, ^#^ *P* < 0.05 and ^##^ *P* < 0.01. Note: THP: tetrahydropalmatine; ox-LDL: oxidized low-density lipoprotein; VSMCs: vascular smooth muscle cells; MDA: malondialdehyde; SOD: superoxide dismutase; ROS: reactive oxygen species; DCFH-DA: 2′,7′-Dichlorofluorescein diacetate; α-SMA: α-smooth muscle actin; SM-MHC: smooth muscle myosin heavy chain; OPN: osteopontin.

U46619, the RhoA pathway agonist, was used to verify the mechanism of THP on RhoA/ROCK1 signaling pathway in ox-LDL-incubated VSMCs. Test of MDA and SOD content was performed with kits (Figure 7a and b). Compared with the ox-LDL group, THP lessened MDA content and enhanced SOD content (*P* < 0.01), while U46619 reversed the above effects (*P* < 0.01 or *P* < 0.05). Measurement of ROS content was made by the DCFH-DA fluorescent probe as displayed in Figure 7c and d. The ox-LDL group exhibited higher ROS content than control cells (*P* < 0.01). There was decreased ROS content in ox-LDL + THP group than in ox-LDL group, with an increase in ROS content in U46619 group than in ox-LDL + THP group (*P* < 0.01). Moreover, detection of phenotypic switching protein expression was done by western blot assay (Figure 7e–h). The elevation of α-SMA and SM-MHC and decline of OPN via THP treatment in ox-LDL-incubated VSMCs was reversed by U46619 intervention (*P* < 0.01 or *P* < 0.05).

## 4 Discussion

In the present study, we found that THP treatment increased cell viability, MMP, and mitochondrial respiration and inhibited cell migration, oxidative stress, ROS content, and apoptosis through down-regulation of the RhoA/ROCK1 signaling pathway in ox-LDL-incubated VSMCs, thereby decelerating AS development. Our study revealed the effects of THP on VSMCs and its molecular mechanism on mitochondrial dysfunction, providing a new drug target, RhoA/ROCK1 signaling pathway, and protective mechanism for the study of AS treatment.

As a CVD caused by the proliferation of VSMCs and cholesterol accumulation, AS seriously threats human life and health [22]. Antiplatelet drugs, statins, nitrates have urged us to find more efficient and safe natural anti-AS drugs from herbal due to their side effects [23]. The antioxidant and anti-apoptosis effects of THP have been confirmed to its cardio-cerebrovascular protection [17]. It has been indicated that THP exerts protective effects on mitochondrial function via inhibiting the decrease in intracellular MMP [24]. Thus, THP has the potential to ameliorate AS and may exert anti-AS activity by enhancing mitochondrial function, inhibiting apoptosis and oxidative stress.

As a vital factor in AS pathogenesis, ox-LDL causes pathological changes and damage [25]. In addition, ox-LDL promotes the formation of fatty plaques and causes degeneration of VSMCs [26], which result in AS. So, we used it as AS models *in vitro*. Oxidative stress has been regarded as a critical mechanism in AS [27], and overproduction of ROS is integral in the occurrence and development of AS. ROS mediates various signaling pathways to participate in the course of AS [28]. In this study, THP intervention reduced MDA and ROS content, while increased SOD activity suggest its antioxidant ability on ox-LDL-incubated VSMCs. In addition, AS development is often accompanied by cell apoptosis [29]. TUNEL staining and Western blot results demonstrated that THP restrained the protein expression of apoptosis-enforcers and promoted the expression of apoptosis-suppressor to reduce apoptosis which suggested that the protection effects of THP on AS may be related to the inhibition on cell apoptosis.

Proliferation and migration of VSMCs are happening during AS [30]. Mitochondria participates in aerobic respiration, as the sites of oxidative metabolism in eukaryotes [5]. OCR represents the rate at which oxygen is consumed by respiration and can indirectly indicate whether mitochondrial function is normal [31]. Mitochondrial dysfunction may cause abnormal respiration. It is reasonable to assume that THP can protect mitochondrial function via regulating the migration of VSMCs and adjusting the OCR. Seahorse XF has been widely utilized to measure mitochondrial function and cell metabolism, involving metabolic pathways such as glycolysis and oxidative phosphorylation [32]. As exhibited in flow cytometry and wound scratch results, THP could regulate the cell cycle and weaken the lateral migration of VSMCs. Moreover, THP increased the decrease trend of MMP caused by ox-LDL intervention, and regulated the mitochondrial respiratory function of VSMCs. These results suggest that THP may increase the MMP and regulate oxygen consumption to protect mitochondrial function on VSMCs and astrict VSMCs migration.

As mentioned above, AS can cause the overproduction of ROS, which promotes the activation of RhoA/ROCK1 signaling pathway [27,33]. It has been indicated that THP has a certain regulatory effect on mitochondrial dysfunction [34] and there are some reasons to consider that the regulation of RhoA/ROCK1 signaling pathway-related protein maybe a new target for THP in the improvement of mitochondrial function in AS [11]. In our study, THP caused inhibition of RhoA/ROCK1 signaling pathway to maintain mitochondrial function for prevention of AS deterioration.

Since AR is an important pathological basis of AS, it is reasonable to speculate that THP may treat AS by influencing the expressions of phenotypic switching proteins in VSMCs [35]. Detection of western blot demonstrated that THP led to increased α-SMA and SM-MHC expression while decreased OPN expression. Moreover, the application of agonist of RhoA/ROCK1 signaling pathway, U46619 [36], further supported the speculation. The effects of THP were reversed by treatment of U46619. Combined with the previous experimental results, it is suggested that THP reduces oxidative stress injury by regulating the expression of key proteins in RhoA/ROCK1 signaling pathway.

There are still some limitations in this study. This work does not further study the upstream and downstream molecular mechanisms of RhoA/ROCK1 signaling pathway by which THP regulates mitochondrial function. Additionally, we only detected the expressions of cell phenotypic switching proteins α-SMA, SM-MHC, and OPN, and did not conduct in-depth studies on their specific sites in VSMCs and its relationship with mitochondrial function. In future study, mitochondrial genome sequencing may be a profound method to further explore the protection effects on mitochondrial function of THP against AS.

## 5 Conclusion

In conclusion, our experimental results confirmed that the role of THP for improving mitochondrial function in AS may relate to the inhibition of RhoA/ROCK1 signaling pathway, offering scientific basis for the clinical application of THP in AS treatment.

## Abbreviations

AR: arterial remodeling
AS: atherosclerosis
ATP: adenosine triphosphate
Bax: Bcl2-associated X
Bcl-2: B-cell lymphoma-2
CCK-8: cell counting kit-8
CVD: cardiovascular disease
DCFH-DA: 2′,7′-dichlorofluorescein diacetate
ELISA: enzyme linked immunosorbent assay
FCCP: carbonyl cyanide phospho-(*p*)-trifluoromethoxy phenylhydrazone
MDA: malondialdehyde
MMP: mitochondrial membrane potential
OCR: oxygen consumption rate
OPN: osteopontin
ox-LDL: oxidized low-density lipoprotein
Rho: Ras homolog gene family
ROCK: Rho-associated protein kinase
ROS: reactive oxygen species
SM-MHC: smooth muscle myosin heavy chain
SOD: superoxide dismutase
THP: tetrahydropalmatine
TUNEL: terminal deoxynucleotidyl transferase-mediated dUTP nick end labeling assay
VSMCs: vascular smooth muscle cells
α-SMA: α-smooth muscle actin
